# Prognostic Value of a Glycolytic Signature and Its Regulation by Y-Box-Binding Protein 1 in Triple-Negative Breast Cancer

**DOI:** 10.3390/cells10081890

**Published:** 2021-07-26

**Authors:** Yi-Wen Lai, Wen-Jing Hsu, Wen-Ying Lee, Cheng-Hsun Chen, Ying-Huei Tsai, Jia-Zih Dai, Ching-Chieh Yang, Cheng-Wei Lin

**Affiliations:** 1 Department of Biochemistry and Molecular Cell Biology, School of Medicine, College of Medicine, Taipei Medical University, Taipei 11031, Taiwan; m120109002@tmu.edu.tw (Y.-W.L.); m120107025@tmu.edu.tw (W.-J.H.); b614105024@tmu.edu.tw (C.-H.C.); b101106067@tmu.edu.tw (Y.-H.T.); d119109007@tmu.edu.tw (J.-Z.D.); 2 Graduate Institute of Medical Sciences, College of Medicine, Taipei Medical University, Taipei 11031, Taiwan; 3 Chi Mei Medical Center, Department of Cytopathology, Tainan 710, Taiwan; d940232@mail.chimei.org.tw; 4 Chi Mei Medical Center, Department of Radiation Oncology, Tainan 710, Taiwan; 5 Department of Pharmacy, Chia-Nan University of Pharmacy and Science, Tainan 717, Taiwan; 6 Cell Physiology and Molecular Image Research Center, Wan Fang Hospital, Taipei Medical University, Taipei 11696, Taiwan; 7 Drug Development and Value Creation Research Center, Kaohsiung Medical University, Kaohsiung 807, Taiwan

**Keywords:** EMT, glycolysis, YBX1, triple-negative breast cancer

## Abstract

Triple-negative breast cancer (TNBC) is the most malignant subtype of breast cancer as it shows a high capacity for metastasis and poor prognoses. Metabolic reprogramming is one of the hallmarks of cancer, and aberrant glycolysis was reported to be upregulated in TNBC. Thus, identifying metabolic biomarkers for diagnoses and investigating cross-talk between glycolysis and invasiveness could potentially enable the development of therapeutics for patients with TNBC. In order to determine novel and reliable metabolic biomarkers for predicting clinical outcomes of TNBC, we analyzed transcriptome levels of glycolysis-related genes in various subtypes of breast cancer from public databases and identified a distinct glycolysis gene signature, which included *ENO1, SLC2A6, LDHA, PFKP, PGAM1*, and *GPI*, that was elevated and associated with poorer prognoses of TNBC patients. Notably, we found a transcription factor named Y-box-binding protein 1 (YBX1) to be strongly associated with this glycolysis gene signature, and it was overexpressed in TNBC. A mechanistic study further validated that YBX1 was upregulated in TNBC cell lines, and knockdown of YBX1 suppressed expression of those glycolytic genes. Moreover, YBX1 expression was positively associated with epithelial-to-mesenchymal transition (EMT) genes in breast cancer patients, and suppression of YBX1 downregulated expressions of EMT-related genes and tumor migration and invasion in MDA-MB-231 and BT549 TNBC cells. Our data revealed an YBX1-glycolysis-EMT network as an attractive diagnostic marker and metabolic target in TNBC patients.

## 1. Introduction

Breast cancer is the most commonly diagnosed malignancy in women, and breast cancer is divided into luminal, human epidermal receptor 2 (HER2)-enriched, and triple-negative breast cancer (TNBC) subtypes, according to the presence of distinct molecular markers, including the estrogen receptor (ER), progesterone receptor (PR), and HER2. Among these, TNBC, which is characterized by the absence of the ER, PR, and HER2, is the most malignant subtype of breast cancer [1,2]. Due to a deficiency of efficient therapeutic targets and its aggressive features, such as resistance to chemotherapy, higher invasiveness, and a strong tendency to metastasize, patients with TNBC have poorer survival outcomes than non-TNBC patients [3,4]. Thus, exploring novel biomarkers is urgently needed to improve the accuracy of diagnoses and efficacy of treatments for TNBC patients.

Reprogramming of cellular metabolism is an emerging hallmark of cancer that provides tumor cells with several advantages for growth, metastasis, resistance to therapeutics, and even survival of the harsh tumor microenvironment (TME) [5,6]. Aerobic glycolysis, also known as the Warburg effect, is predominantly upregulated in a variety of solid tumors, including breast cancer [7]. Notably, it was shown that TNBC cells expressed upregulation of metabolic receptors and a higher glycolytic capacity than non-TNBC cells [8,9], suggesting a higher demand for glucose utilization to support the aggressive features of TNBC cells. However, a reliable glycolysis-associated marker for predicting the prognosis of TNBC patients and the underlying regulatory mechanism that links glycolysis and TNBC invasiveness are still unclear.

In an attempt to investigate the upstream regulator of the glycolysis-associated signature in TNBC, we identified that Y-box-binding protein 1 (YBX1) is overexpressed and associated with glycolysis-related genes in TNBC. YBX1 is a member of the cold-shock domain protein family and is considered the most evolutionarily conserved nucleic acid-binding protein. YBX1 is capable of binding with DNA/RNA, and thus acts as both a transcription factor and a translational regulator [10]. Recently, accumulating reports on the oncogenic function of YBX1 in cancer biology have predominantly associated its transcriptional role of regulating genes with DNA repair, cell adhesion, angiogenesis, and drug resistance [11,12,13,14], thus confirming its therapeutic potential. Overexpression and/or nuclear accumulation of YBX1 can predict poor outcomes in a variety of malignancies, including breast cancer [15,16]. However, the association between YBX1’s ability to regulate glycolysis and the invasiveness of TNBC cells is not clear.

Results presented herein indicate that YBX1 is overexpressed and associated with glycolysis- and epithelial-to-mesenchymal transition (EMT)-related gene signatures in TNBC patients. Gain-of-function and loss-of-function studies further validated that YBX1 regulated glycolysis- and EMT-related gene expressions and was crucial for tumor invasiveness, thus indicating that YBX1 exhibits clinical diagnostic value and is a potential metabolic target for TNBC.

## 2. Materials and Methods

### 2.1. Cell Culture

Human breast cancer cell lines, including the BT549, MDA-MB-231, HCC1806, MCF7, and T47D cell lines, were purchased from the American Type Culture Collection (ATCC, Manassas, VA, USA) or Bioresource Collection Research Center (BCRC, Hsinchu, Taiwan). Cells were cultured at 37 °C in a humidified atmosphere of 5% CO_2_. MDA-MB-231 cells were maintained in Dulbecco’s modified Eagle medium (DMEM), and BT549 cells were cultured in Roswell Park Memorial Institute (RPMI) 1640 medium. Culture media were supplemented with 7% fetal bovine serum (FBS), 1% antibiotic-antimycotic, and 1% Glutagro. All cell culture supplements were purchased from Corning (New York, NY, USA). A lactate assay kit was purchased from Abcam (Cambridge, MA, USA).

### 2.2. Transwell Migration and Invasion Assay

MDA-MB-231 and BT549 cells were seeded at a density of 5 × 10^4^ cells per chamber in an 8-µm-pore size transwell insert (Corning) coated with or without Matrigel (BD Biosciences, Franklin Lakes, NJ, USA). Serum-free DMEM was added to the upper chamber, and DMEM containing 7% FBS was added to the lower chamber as the chemoattractant. Cells were incubated for 24 h, fixed in ice-cold methanol, and stained with a 1% crystal violet solution. Matrigel and any unmigrated cells were removed using cotton swabs prior to observation. For the wound-healing assay, cells were seeded in six-well plates and scratched with a 200 μL pipette tip. Culture media were refreshed to remove detached cells, and cell migration into the wound area was observed under an inverted microscope. Quantification of wound area was performed by using Image J software, version 1.51 (NIH, Betesda, MD, USA) [17]. 

### 2.3. Plasmids and Transfection

Human YBX1 small hairpin (sh)RNA#1 (TRCN0000007948) and shRNA#2 (TRCN0000007952) in pLKO.puro vectors were obtained from the National RNAi Core Facility (Academia Sinica, Taipei, Taiwan). Target sequences of shRNA#1 and shRNA#2 were GCTTACCATCTCTACCATCAT and CCAGTTCAAGGCAGTAAATAT, respectively. Lentiviral preparation and viral infection were performed as previously described. In brief, the pLKO.shRNA, pCMV-∆R8.91, and pMDG plasmids were cotransfected into 293T cells using the PolyJet transfection reagent (SignaGen Laboratories, Ijamsville, MD, USA). After transfection, culture media were refreshed and replaced with complete medium for another 48 h. MDA-MB-231 and BT549 cells were seeded in six-well plates and mixed with virus-containing supernatants with polybrene (8 μg/mL) and incubated for another 96 h. shRNA targeting LacZ was used as a negative control shRNA [18].

### 2.4. Western Blotting

Cell lysates were collected using radioimmunoprecipitation assay (RIPA) buffer supplemented with a protease and phosphatase inhibitor cocktail (Roche, Mannheim, German). Approximately 30~50 μg of proteins was separated in denaturing sodium dodecylsulfate (SDS)-polyacrylamide gel electrophoresis (PAGE) and transferred to polyvinylidene difluoride (PVDF) membranes (Millipore, Bedford, MA, USA). After blocking with 1% bovine serum albumin/TBST blocking buffer, the membranes were incubated overnight with specific primary antibodies. Membranes were washed three times with the TBST wash buffer and probed with an appropriate horseradish peroxidase-conjugated secondary antibody (Jackson ImmunoResearch, West Grove, PA, USA). Protein bands were detected with an enhanced chemiluminescence (ECL) reagent (Millipore). A primary antibody for YBX1 was purchased from Santa Cruz Biotechnology (Santa Cruz, CA, USA). Antibodies for α-tubulin, E-cadherin, vimentin, Twist, Slug, and N-cadherin were purchased from GeneTex (San Antonio, TX, USA). Western blotting was performed at least three times, and representative experiments are shown.

### 2.5. Real-Time Quantitative Polymerase Chain Reaction (qPCR)

Total RNA was extracted with a GENzolTM TriRNA Pure kit (Geneaid, Taipei, Taiwan). The RNA concentration was measured with Nanodrop 2000 (Thermo Scientific, Waltham, MA, USA), and 1 µg of total RNA was used to generate complementary (c)DNA with Oligo(dT)_16_ and M-MLV reverse transcriptase (Promega, Madison, WI, USA) and amplified with GoTaq qPCR Master Mix (Promega) in a StepOne Plus Real-Time PCR system (Applied Biosystems, Darmstadt, Germany). Primer sequences are shown in Supplementary Table S1. 18s was used as a reference gene. All reverse-transcription (RT)-qPCRs were carried out in triplicate. Results were calculated using the ΔΔCT equation and are expressed as multiples of change relative to a control group [19].

### 2.6. Data Acquisition

The raw gene expression data of the YBX family, and glycolysis- and EMT-related genes obtained by RNA sequencing (RNA-Seq), along with clinical data were downloaded from The Cancer Genome Atlas Breast Invasive Carcinoma (TCGA_BRCA) dataset (https://www.cbioportol.org, accessed on 30 March 2021). Data were generated using the mRNA expression z-scores relative to all samples (log RNA Seq V2 RSEM). Cell line data were downloaded from the Cancer Cell Line Encyclopedia (CCLE) (https://portals.broadinstitute.org/ccle, accessed on 5 May 2021), and units of mRNA in the CCLE were log2-normalized RNA expressions. Breast cancer cohorts including GSE18864, GSE21653, and GSE76275 were downloaded from Gene Expression Omnibus (GEO) databases [20,21,22,23,24]. Recurrence-free survival (RFS) prognoses of YBX1 and glycolysis genes in luminal, HER2, and basal-like TNBC breast cancer patients were analyzed using the KM plotter (https://kmplot.com, accessed on 5 May 2021). High- and low-expression groups of patients were stratified according to optimal gene expression cutoff values. Analyses of associations of YBX1 with glycolysis and cellular metabolism were carried out using a gene set enrichment analysis (GSEA) algorithm with Hallmark gene sets (https://www.gsea-msigdb.org, accessed on 15 January 2021).

### 2.7. Immunochemistry Analyses

Breast cancer tissue microarrays were purchased from US Biomax, Inc. (Rockville, MD, USA). Deparaffinized tissue slides were rehydrated with different concentrations of alcohol and washed with dH_2_O. Antigen was retrieved with citrate unmasking buffer (Vector Laboratories, Burlingame, CA, USA), and blocked with 1% bovine serum albumin (BSA). Slides were then probed with anti-YBX1 antibody (Santa Cruz Biotechnology) overnight in a humidity chamber and further incubated with SignalStain^®^ Boost IHC detection reagent (Cell Signaling Technology, Danvers, MA, USA). Protein expression was visualized with a 3, 3-diaminobenzidine (DAB) peroxidase substrate (Vector Laboratories). The strong and moderate staining were defined as high expression, and weak and nil staining were defined as low expression. 

### 2.8. Statistical Analyses

Values are expressed as the mean ± standard deviation (SD) of three independent experiments. Statistical significance was determined by an unpaired, two-tailed Student’s *t*-test unless otherwise specified. A correlation coefficient was analyzed by Pearson’s test. * *p* < 0.05, ** *p* < 0.01, and *** *p* < 0.001 were regarded as significant. All statistical analyses were carried out using GraphPad Prism v6.0 software.

## 3. Results

### 3.1. Identifying a Distinct Glycolysis Gene Signature in TNBC

**Figure 1:**
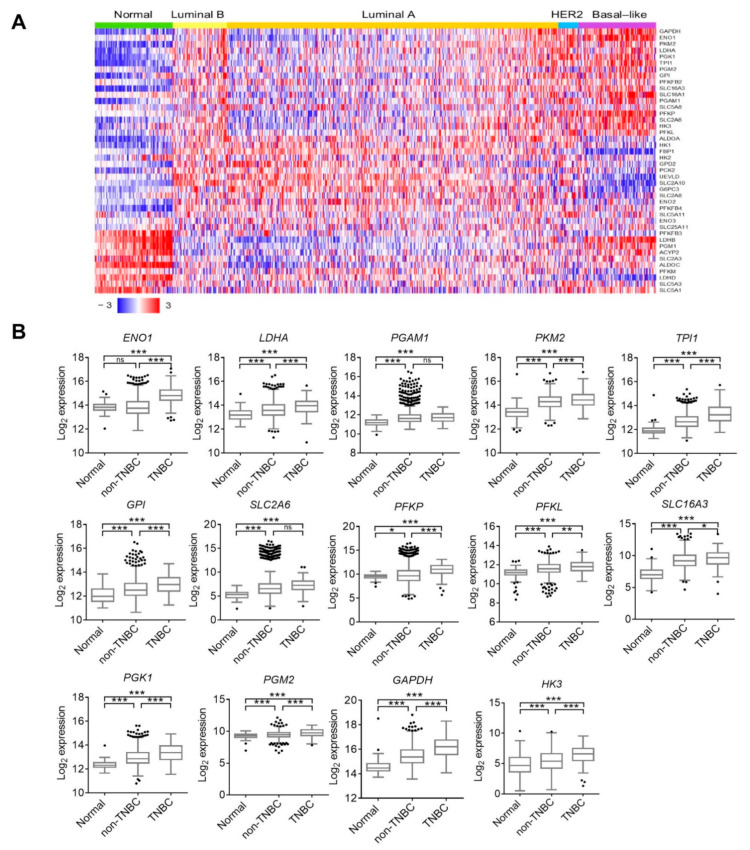
Transcriptome levels of glycolysis-related genes in breast cancer patients. (**A**) Heat map plot of 42 glycolysis-related gene expressions using TCGA_BRCA dataset. Breast cancer patients were characterized by molecular subtypes, including luminal A (*n* = 499), luminal B (*n* = 197), human epidermal receptor 2 (HER2) (*n* = 78), and basal-like triple-negative breast cancer (TNBC) (*n* = 171), and normal adjacent tissues (*n* = 36). (**B**) Box plots with Tukey whiskers of 14 glycolysis-related gene expressions in TCGA_BRCA cohort. Subjects were characterized as normal (*n* = 36), basal-like TNBC (*n* = 171), and non-TNBC (*n* = 774). * *p* < 0.05, ** *p* < 0.01, and *** *p* < 0.001 as determined by an ANOVA. ns, not significant.

**Figure 2:**
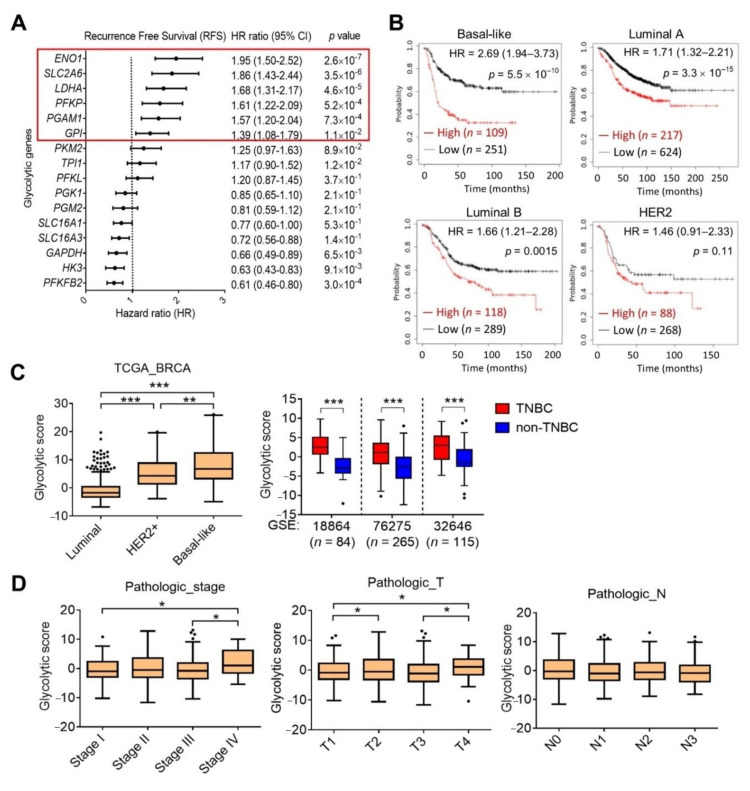
Upregulation of the glycolysis signature confers a poor prognosis in triple-negative breast cancer patients. (**A**) Forest plot showing hazard ratio (HR) estimates and 95% confidence intervals (CI) of 14 glycolysis-related genes and recurrence-free survival in breast cancer patients. The six selected genes as a glycolysis signature are shown in red. (**B**) Kaplan-Meier curve analysis of recurrence-free survival probability of breast cancer patients stratified by glycolysis signature expression. (**C**) Expression levels of the glycolysis signature in breast cancer patients according to TCGA and GEO (GSE18864, GSE76275, and GSE32646) datasets. ** *p* < 0.01, and *** *p* < 0.001 as determined by unpaired *t*-tests. (**D**) Box plot showing the glycolysis signature score grouped by pathologic stage, tumor grade, and lymph node status. * *p* < 0.05, ** *p* < 0.01, and *** *p* < 0.001 as determined by an ANOVA.

To identify a distinct glycolysis gene signature in TNBC, we examined transcriptome levels of 42 glycolysis-related genes from a TCGA breast-invasive carcinoma (BRCA) dataset which were stratified by molecular subtypes, including normal (*n* = 36), luminal A (*n* = 499), luminal B (*n* = 197), HER2 (*n* = 78), and basal-like TNBC (*n* = 171) (Figure 1A). We selected 14 genes which were significantly upregulated in TNBC compared to non-TNBC or normal tissues (Figure 1B). RFS from the KM plotter database was used to further examine prognostic correlations with selected genes. Among these, we selected six genes, including enolase 1 (*ENO1*), solute carrier family 2 member 6 (*SLC2A6*), lactate dehydrogenase A (*LDHA*), phosphofructokinase, platelet (*PFKP*), phosphoglycerate mutase 1 (*PGAM1*), and glucose-6-phosphate isomerase (*GPI*), that were individually associated with poor RFS in breast cancer patients (Figure 2A), and their overexpressions specifically rendered the worst survival probability in patients with TNBC (hazard ratio (HR) = 2.69, *p* < 0.0001), compared to the luminal A (HR = 1.71, *p* < 0.0001), luminal B (HR = 1.66, *p* = 0.0015), and HER2 subtypes (HR = 1.46, *p* = 0.11) (Figure 2B). We defined these six genes as a glycolysis signature, and validated its overexpression in basal-like TNBC, compared to HER2 and luminal subtypes using TCGA_BRCA dataset (Figure 2C). In support of the above results, we analyzed three other breast cancer cohort datasets from the GEO, viz., GSE18864 (*n* = 84), GSE76275 (*n* = 265), and GSE32646 (*n* = 115), and also confirmed upregulation of the glycolysis signature in TNBC compared to non-TNBC samples (Figure 2C). Moreover, breast cancer patients with elevated expression of the glycolytic signature were associated with advanced pathologic stages and tumor grades, but not with nodal stages (Figure 2D). These results suggest a specific glycolysis gene signature for predicting disease progression and survival probability of TNBC patients.

### 3.2. Association of YBX1 with the Glycolysis Signature

**Figure 3:**
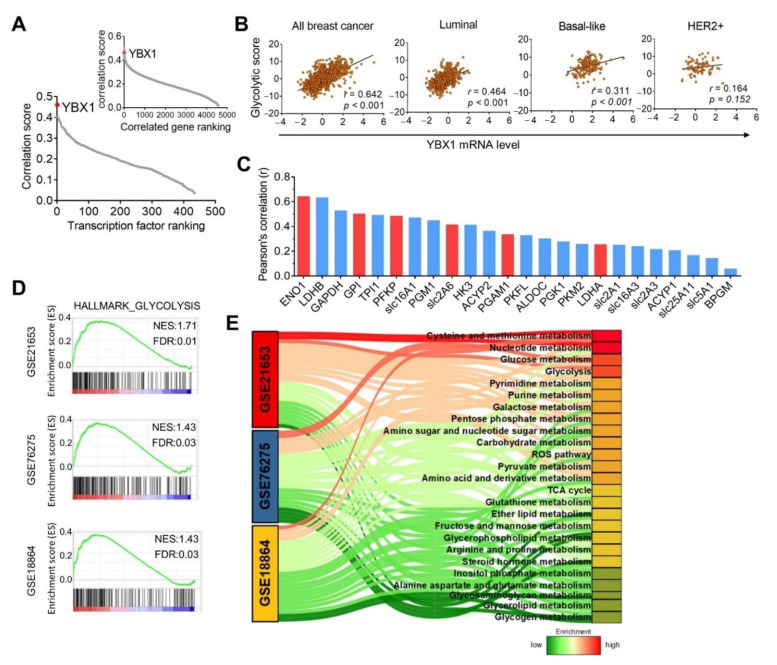
YBX1 is correlated with the glycolysis signature in triple-negative breast cancer. (**A**) Ranked abundance plots of transcription factors and transcripts (insert plot) with the glycolysis signature according to TCGA_BRCA dataset. (**B**) Scatterplot of Pearson’s correlation analysis of YBX1 and the glycolysis signature using TCGA_BRCA dataset. (**C**) Positive correlations of YBX1 and glycolysis-related genes, as analyzed by Pearson’s correlations. Red colors denote the glycolytic signature. (**D**) Representative GSEA plots showing the association between YBX1 expression and glycolysis in the GSE18864, GSE21653, and GSE76275 datasets. (**E**) Sankey diagram showing enrichment of YBX1 with metabolic processes in the GSE18864, GSE21653, and GSE76275 datasets.

To unravel the upstream regulator indicated by the glycolysis signature, we screened 4557 genes which showed positive correlations with the glycolysis signature from TCGA_BRCA dataset, and tentative transcriptional factors were further filtered from among those 4557 genes. Among these, Y-box-binding protein 1 (YBX1) showed the most significant correlation with the glycolysis signature (Figure 3A). Additionally, YBX1 mRNA levels showed positive correlations with the glycolysis signature in TNBC (Pearson’s *r* = 0.311, *p* < 0.001) and luminal (Pearson’s *r* = 0.464, *p* < 0.001) subtypes, but not in HER2-enriched subtypes (Pearson’s *r* = 0.164, *p* = 0.152) (Figure 3B). Moreover, YBX1 expression was not only correlated with the glycolysis signature, it was also associated with other glycolysis-related genes (Figure 3C). We further performed a gene set enrichment analysis (GSEA), and results showed that YBX1 overexpression was positively associated with the glycolysis hallmark using GEO breast cancer cohorts (Figure 3D). Moreover, a Sankey diagram analysis revealed that several metabolic processes, including cysteine and methionine metabolism, nucleotide metabolism, glucose metabolism, galactose metabolism, and the pentose phosphate pathway, were significantly associated with YBX1 expression levels (Figure 3E). These data suggest that YBX1 might play a role in regulating cancer metabolism.

### 3.3. YBX1 Is Overexpressed in TNBC and Is a Prognostic Marker

**Figure 4:**
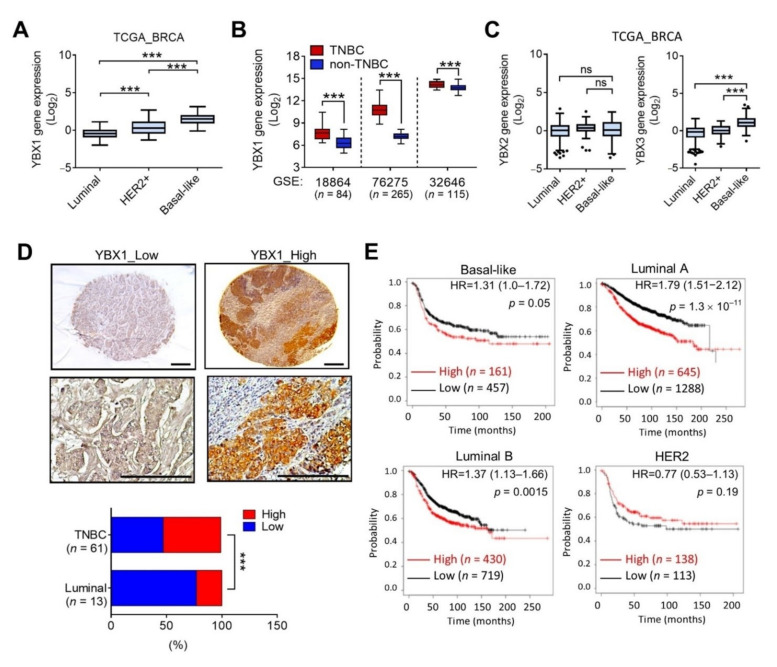
Upregulation of YBX1 in triple-negative breast cancer. (**A**) Box plots with Tukey whiskers of YBX1 mRNA levels in TCGA_BRCA cohort. *** *p* < 0.001 as determined by an ANOVA. (**B**) Box plots showing expression levels of YBX1 in TNBC and non-TNBC samples in GEO (GSE18864, GSE76275, and GSE32646) datasets. *** *p* < 0.001 as determined by unpaired *t*-tests. (**C**) Expression levels of YBX2 and YBX3 in luminal, human epidermal receptor 2 (HER2), and basal-like TNBC samples according to TCGA_BRCA dataset. *** *p* < 0.001 as determined by an ANOVA. ns, not significant. (**D**) Representative images of immunohistochemical staining of YBX1 protein levels in breast cancer tissues (upper panel). Scale bar = 100 μm. Statistical analysis of the association of YBX1 with TNBC and luminal breast cancer was performed using Chi-squared tests (lower panel). (**E**) Kaplan-Meier curve analysis of YBX1 expression levels and recurrence-free survival probabilities in luminal, HER2, and TNBC patients.

We further explored YBX1 mRNA levels using TCGA_BRCA dataset, and results showed that YBX1 was significantly upregulated in TNBC compared to the HER2 (*p* < 0.001) and luminal subtypes (*p* < 0.001) (Figure 4A). Similar results were observed in other breast cancer cohorts in which YBX1 was elevated in TNBC compared to non-TNBC patients (Figure 4B). Additionally, we analyzed mRNA levels of YBX family members including YBX2 and YBX3 in breast cancer patients, and results showed that YBX2 mRNA levels showed no difference between TNBC and non-TNBC patients, while YBX3 was upregulated in TNBC patients compared to the non-TNBC group (Figure 4C). To validate the expression of YBX1 in breast cancer, we performed immunohistochemistry. As shown in Figure 4D, YBX1 was elevated in 33 (54%) of 61 TNBC cases and 5 (38%) of 13 luminal cases, indicating its upregulation in TNBC (Figure 4D). Moreover, the Kaplan–Meier analysis showed that YBX1 overexpression rendered an unfavorable RFS probability in the TNBC (HR = 1.31, *p* = 0.05) and luminal subtypes (HR = 1.79, *p* < 0.001 for luminal A and HR = 1.37, *p* = 0.0015 for luminal B), but not in the HER2 subtype (Figure 4E).

### 3.4. YBX1 Regulates Glycolysis Signature Expression

**Figure 5:**
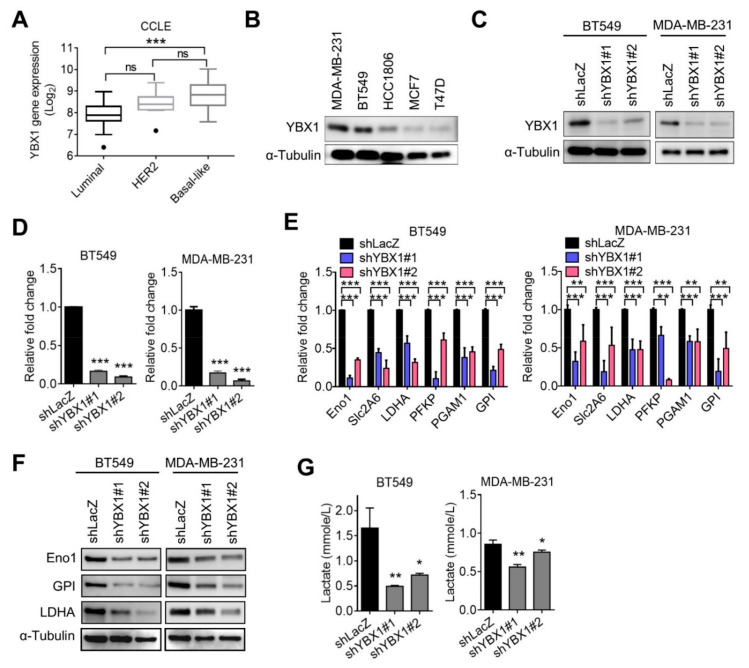
YBX1 regulated glycolysis genes expression. (**A**) Expression levels of YBX1 in luminal, HER2, and TNBC cell lines according to the CCLE breast cancer dataset. *** *p* < 0.001 as determined by an ANOVA. ns, not significant. (**B**) Western blot analysis of YBX1 protein levels in breast cancer cell lines. (**C** and **D**) Knockdown efficiencies of YBX1 shRNAs in MDA-MB-231 and BT549 cells, as measured by Western blot and RT-qPCR analyses. (**E**) RT-qPCR analysis of glycolysis signature expression in YBX1-knockdown MDA-MB-231 and BT549 cells. (**F**) Western blot analysis of glycolysis protein levels in YBX1-knockdown MDA-MB-231 and BT549 cells. (**G**) Production of lactate production in YBX1-knockdown MDA-MB-231 and BT549 cells, as measured by a lactate assay kit. * *p* < 0.05, ** *p* < 0.01, and *** *p* < 0.001 as determined by unpaired *t*-tests.

To gain insights into the significance of YBX1 in breast cancer, we analyzed YBX1 levels using the CCLE database, and results were consistent with clinical data that YBX1 was upregulated in TNBC compared to non-TNBC cell lines (Figure 5A). Results of Western blotting further confirmed that the YBX1 protein level was overexpressed in TNBC cell lines including MDA-MB-231 and BT549 cells (Figure 5B). We further knocked-down YBX1 in MDA-MB-231 and BT549 cells and validated the knockdown efficiency by Western blot and real-time PCR analyses (Figure 5C,D). Importantly, suppression of YBX1 significantly downregulated expression of the glycolysis signature in both MDA-MB-231 and BT549 cells (Figure 5E). Results of Western blotting further confirmed downregulation of ENO1, GPI, and LDHA protein levels in YBX1-knockdown MDA-MB-231 and BT549 cells (Figure 5F). Moreover, suppression of YBX1 significantly decreased lactate secretion (Figure 5G), indicating that YBX1 plays a crucial role in regulating glycolysis in TNBC cells.

### 3.5. YBX1 Is Associated with the EMT Gene Expressions and Invasiveness of Breast Cancer

**Figure 6:**
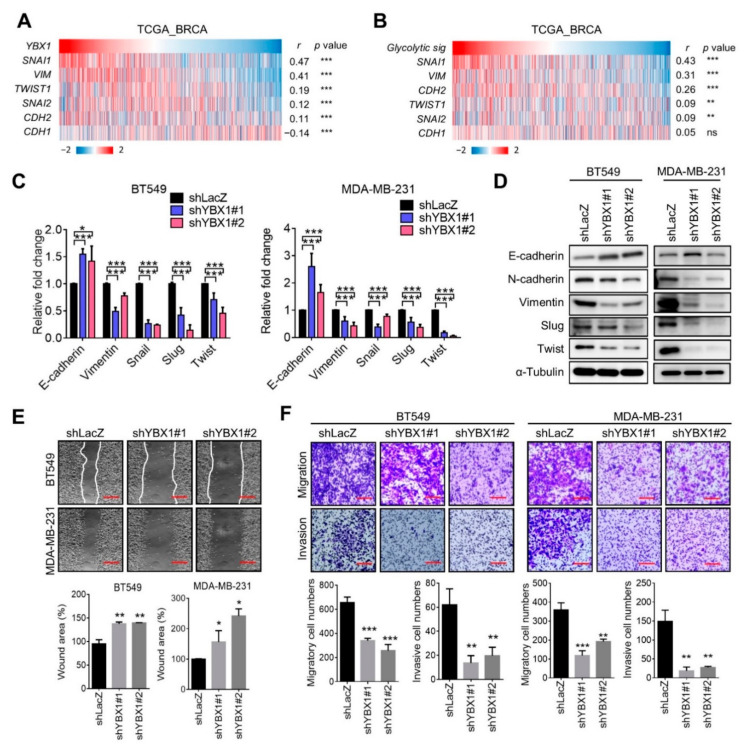
YBX1 promotes epithelial-to-mesenchymal transition gene expressions and invasiveness of triple-negative breast cancer cells. (**A**) Heat map plot showing correlations between YBX1 and EMT-related genes in TCGA_BRCA dataset. (**B**) Heat map plot showing correlations between the glycolysis signature and EMT-related genes in TCGA_BRCA dataset. ns, not significant. (**C**) RT-qPCR analyses of EMT-related gene expressions in YBX1-knockdown BT549 and MDA-MB-231 cells. (**D**) EMT-related protein levels in YBX1-knockdown BT549 and MDA-MB-231 cells. (**E**) Wound-healing assay of cell migration using YBX1-knockdown BT549 and MDA-MB-231 cells. Wound area was quantified and expressed as percentage relative to the control group. Scale bar = 100 μm. (**F**) Transwell analyses of migration and invasion with YBX1-knockdown MDA-MB-231 and BT549 cells. Representative images from randomly selected fields of the transwell insert are shown. Scale bar = 100 μm. Error bars indicate the mean ± SD of at least three independent replicates. * *p* < 0.05; ** *p* < 0.01; *** *p* < 0.001, as determined by unpaired *t*-tests.

To further evaluate the molecular function of YBX1 in promoting breast tumor progression, we analyzed associations between YBX1 and EMT genes. As shown in Figure 5A, YBX1 expression was positively correlated with EMT genes, including Snail (*SNAI1*), vimentin (*VIM*), N-cadherin (*CDH2*), Twist1 (*TWIST1*), and Slug (*SNAI2*), whereas it was negatively correlated with E-cadherin (*CDH1*) (Figure 6A).

Additionally, the glycolysis signature showed positive correlations with mesenchymal-related genes in breast cancer patients (Figure 6B), suggesting that crosstalk between glycolysis and the EMT may be regulated by YBX1. In support of these observations, we found that suppression of YBX1 substantially downregulated EMT gene expressions in BT549 and MDA-MB-231 cells. Consistent results were obtained from Western blot analyses of YBX1-knockdown inhibiting mesenchymal-associated protein levels (Figure 6C,D). To assess the effect of YBX1 on tumor mobility, a wound-healing assay was carried out with YBX1-knockdown MDA-MB-231 and BT549 cells. As shown in Figure 6E, suppression of YBX1 reduced tumor cell migration into the wound (Figure 6E). The transwell analysis also showed that YBX1-knockdown significantly suppressed the migratory and invasive capabilities (Figure 6F). These findings indicate that YBX1 upregulates EMT gene expressions and promotes invasiveness of TNBC.

## 4. Discussion

Patients with TNBC showed the worst prognoses compared to the other breast cancer subtypes. TNBC exhibits higher invasive and glycolysis features than non-TNBC, and identifying reliable biomarkers with predictive prognostic value is important for TNBC patients. In the present study, a glycolysis gene signature that may act as a prognostic biomarker for patients with TNBC was evaluated. Moreover, we identified that YBX1 acts as an upstream regulator to regulate glycolysis and EMT gene expressions. YBX1 is elevated in a variety of malignancies, including breast cancer; however, the prognostic role of YBX1 in TNBC is unclear. We identified that YBX1 was overexpressed in TNBC cells, whereas suppression of YBX1 in TNBC cells downregulated glycolytic and EMT gene expressions, and this was accompanied by inhibition of tumor migration and invasion. Our data showed that YBX1 overexpression was associated with poor survival outcomes in TNBC and luminal subtype breast cancers. However, YBX1 was expressed at lower levels in the luminal subtypes compared to HER2-positive or TNBC breast cancer. It should be noted that the glycolysis signature was associated with poor prognoses in luminal and basal-like breast cancer patients, but not in the HER2 subtype, indicating that HER2-enriched breast cancer may have distinct molecular characteristics which are independent of YBX1 or glycolysis. These findings highlight the prognostic value of YBX1 and the glycolysis signature in TNBC patients and suggest its potential as a therapeutic target.

The EMT is an evolutionary biological process in both tissue development and tumor metastasis. YBX1 was reported to promote the EMT in breast, melanoma, and nasopharyngeal carcinomas [25,26,27]. Accordingly, hypoxia induced the association of YBX1 with zinc-finger E-box-binding homeobox 1 (ZEB1), a key regulator of the EMT, to promote metastasis of pancreatic cancer [28]. Additionally, translational regulation of hypoxia-inducing factor 1α (HIF1α) by YBX1 promoted sarcoma metastasis [29]. Moreover, the concomitant expressions of YBX1 and HIF1α elevated the glycolytic capacity of bladder cancer cells [30]. Because HIF1α is pivotal to inducing EMT and glycolysis gene expressions [31,32], these data suggest that YBX1 may play a role in promoting the HIF1α-mediated EMT and glycolysis and also indicate that YBX1 is a key component in linking the EMT and glycolysis. Previous studies reported that suppression of YBX1 inhibited the invasiveness of TNBC cells through downregulating phosphatidylinositol 3-kinase (PI3K) and mitogen-activated protein kinase (MAPK) signaling and reducing expressions of metalloproteinase-1 (MMP1) and beta-catenin in TNBC [33]. Additionally, YBX1 participates in paclitaxel resistance in TNBC cells [34], but the downstream targets of YBX1 in governing tumor invasiveness and drug resistance are unclear. In the present study, we found that YBX1 expression was strongly associated with the glycolysis gene signature and concomitantly correlated with EMT genes in TNBC patients. Suppression of YBX1 downregulated EMT- and glycolysis-related gene expressions and inhibited the migration and invasion of TNBC cells. Reprogramming of glycolysis facilitates the EMT, which also plays crucial roles in cancer stemness, metastasis, and drug resistance [35,36]. Previous studies, including ours, reported that overexpression of glucose transporter 3 (GLUT3) promoted the EMT and invasiveness of glioma, colon, and breast cancer cells [37,38,39]. Additionally, ENO1 and LDHA were reported to promote the EMT and stemness in lung and breast cancer cells [40,41], suggesting that upregulation of glycolytic genes by YBX1 plays a crucial role in promoting tumor aggressiveness. In addition, YBX1 transcriptionally regulates a subset of mRNAs involved in the EMT, and YBX1 directly activates cap-independent translation of Snail [27]. Additionally, several studies reported that YBX1 regulates EMT gene expressions by coordinating with noncoding RNAs such as long noncoding (lnc)RNAs or circular (circ)RNAs [25,42,43]. Taken together, these data indicate that YBX1 promotes tumor aggressiveness through either direct regulation of EMT genes or indirect modulation of glycolysis. Therefore, the YBX1-mediated glycolysis-EMT network could serve as a valuable diagnostic marker to identify TNBC patients at risk of developing disease progression.

Aberrant glycolysis is commonly associated with proinflammatory responses and immune evasion in the TME [37,44]. Recent studies showed that YBX1 regulated programmed death ligand 1 (PD-L1) expression to mediate immune escape in prostate cancer [45], and the infiltrating cluster of differentiation 4-positive (CD4+) T cells promoted the growth of renal cell carcinoma via modulating transforming growth factor-β1 (TGFβ1)/YBX1/HIF2α signals [46]. These data indicate that YBX1 may play a role in tumor immunity and also provide therapeutic potential in cancer immunotherapy. However, little is known about the role of YBX1 in regulating the inflammatory TME, and more research is needed to clarify this aspect. In addition to transcriptional regulation by YBX1, it is also worth noting that the RNA-binding capacity of YBX1 through association with lncRNAs and microRNAs has recently garnered much attention due to its regulation of expressions of genes related to the cell cycle, nucleotide metabolism, migration, and cytokines [47,48,49,50]. These findings suggest the importance of YBX1 in tumor development. Taken together, YBX1 is upregulated in TNBC and plays a role in TNBC invasiveness by regulating glycolysis- and EMT-related gene expressions. Moreover, we identified a glycolysis gene signature for predicting prognoses of TNBC patients. The YBX1-glycolysis-EMT network could be used as a reliable diagnostic biomarker and a promising therapeutic approach for TNBC.

Collectively, we identified a glycolytic signature for predicting prognoses of TNBC patients and its regulation by YBX1, providing new insights into the mechanism by which YBX1 contributes to the progression of breast cancer.

## Data Availability

Not applicable.

## Supplementary Materials

The following are available online at https://www.mdpi.com/article/10.3390/cells10081890/s1, Table S1: List of oligonucleotides for the real-time PCR.
