# Supplementary material for: Prognostic Value of a Glycolytic Signature and Its Regulation by Y-Box-Binding Protein 1 in Triple-Negative Breast Cancer
Source: Cells. 2021 Jul 26;10(8):1890. doi: 10.3390/cells10081890 (PMC8392807; doi:10.3390/cells10081890)
Supplement: Supplementary file 1 [file cells-10-01890-s001.zip › cells-1280267-supplementary.pdf]

cells MDPI

Supplementary Material

Table S1. List of oligonucleotides for the real-time PCR.

Gene	Forward Primer (5′ to 3′)	Reverse Primer (5′ to 3′)
ENO1	GCC TCC TGC TCA AAG TCA AC	AAC GAT GAG ACA CCA TGA CG
SLC2A6	GGC TGC TCA TGT CTG AGG TC	GAT GGC CGC GAA GAA GAA GAA
LDHA	GAT TCC AGT GTG CCT GTA TGG	CTA CAG AGA GTC CAA TAG CCC
PFKP	CGC CTA CCT CAA CGT GGT G	ACC TCC AGA ACG AAG GTC CTC
PGAM1	CTC CTA TGA TGT CCC ACC	CTC TGG CAA TAG TAT CCT TC
GPI	GAA GTG CTG GTC CAT CCA GT	AAC ATG TTC GAG TTC TGG GA
E-cadherin	GGA ACT ATG AAA AGT GGG CTT G	AAA TTG CCA GGC TCA ATG AC
Vimentin	TGT CCA AAT CGA TGT GGA TGT TTC	TTG TAC CAT TCT TCT GCC TCC TG
Snail	GCT GCA GGA CTC TAA TCC AGA	ATC TCC GGA GGT GGG ATG
Slug	GCA TTT CAA CGC CTC CAA AAA GCC	AGC AGC GGT AGT CCA CAC AGT GAT
Twist	GGC ATC ACT ATG GAC TTT CTC TAT T	GGC CAG TTT GAT CCC AGT ATT
YBX1	TAG ACG CTA TCC ACG TCG TAG	ATC CCT CGT TCT TTT CCC CAC
β-actin	AAG TCC CTT GCC ATC CTA AAA	ATG CTA TCA CCT CCC CTG TG
